# A novel nanohydroxyapatite/polyamide-66 cage for reducing the subsidence rate after single-level anterior cervical discectomy and fusion: a comparative study of 7-year follow-up

**DOI:** 10.1186/s13018-023-03521-1

**Published:** 2023-01-18

**Authors:** Zhimou Zeng, Ce Zhu, Zhipeng Deng, Limin Liu, Yueming Song

**Affiliations:** 1 Department of Orthopedic Surgery and Orthopedics Research Institute, West China Hospital, Sichuan University, No. 37 Guoxue Road, Chengdu, 610041 Sichuan China; 2 Department of Orthopedic Surgery, The First Affiliated Hospital of Chengdu Medical College, Chengdu, 610500 China

**Keywords:** Nano-hydroxyapatite/polyamide-66, Cage, Subsidence, ACDF

## Abstract

**Background:**

A novel nanohydroxyapatite/polyamide-66 cage (n-HA/PA66 cage) with a horseshoe shape was designed to lower the subsidence rate of the traditional hollow cylindrical n-HA/PA66 cage. However, no studies have compared the incidence of subsidence in the two cages. The purpose of this study was to compare the long-term clinical and radiological outcomes of the novel n-HA/PA66 cage with the hollow cylindrical n-HA/PA66 cage after anterior cervical discectomy and fusion (ACDF) to treat single-level cervical degenerative disk disease (CDDD).

**Methods:**

Fifty-two patients with novel n-HA/PA66 cages (Group A) and fifty-five patients with hollow cylindrical n-HA/PA66 cages (Group B) were included. The radiological parameters included intervertebral height (IH), C2-7 angle (C2-7a), segmental alignment (SA), subsidence rate, and fusion rate. The clinical outcomes were visual analog scale (VAS) scores, Japanese Orthopedic Association (JOA) scores, and patient satisfaction rates.

**Results:**

The pre- and postoperative SA, C2-7a, and fusion rates of the patients in Groups A and B were similar. The preoperative and 6-month postoperative IHs in both groups were comparable. However, the final follow-up IH in Group B was significantly smaller than that in Group A (35.9 mm vs. 36.7 mm). The difference in the subsidence rates at the final follow-up between Group A (5.8%, 3/52) and Group B (18.2%, 10/55) was significant. The VAS score, JOA score, and patient satisfaction rate were not significantly different.

**Conclusions:**

The novel n-HA/PA66 cage had similar favorable SA, C2-7a, fusion rate, and clinical outcomes compared to the hollow cylindrical n-HA/PA66 cage for treating single-level ACDF. Moreover, the novel n-HA/PA66 cage achieved a lower subsidence rate and higher IH than the hollow cylindrical n-HA/PA66 cage at the final follow-up.

## Introduction

With the aging of the population, the incidence of cervical degenerative disk disease (CDDD) is significantly increasing [1, 2]. The main complaints of CDDD are neck pain, myelopathy, and/or radiculopathy, which impair the professional capability and daily life of patients [3].

Robinson and Smith first reported anterior cervical discectomy and fusion (ACDF) in the 1950s, and it evolved to be the most commonly used method for the surgical treatment of CDDD [4, 5]. The surgical procedure of ACDF has been relatively standard over the decades since its introduction. The focus has been for improvements through the alterations of the intervertebral cage with different materials and designs [6].

Currently, Titanium and PEEK are two materials primarily used to produce cervical intervertebral cages. Titanium has good biocompatibility, but it is radiopaque and has a relatively high elastic modulus, which could lead to stress shielding and a high subsidence rate [7]. PEEK can achieve suitable biomechanical support and radiolucency, but its disadvantage is its intrinsic bioinertness [8, 9].

The hollow cylindrical nanohydroxyapatite/polyamide-66 cage (n-HA/PA66, Sichuan National Nano Technology Co., Ltd. Chengdu, Sichuan) is composed of nanohydroxyapatite and polyamide-66, and its biomechanical properties are found to match well with those of natural bone [10, 11]. The high n-HA content in the composites determines the favorable biocompatibility and bioactivity of the n-HA/PA66 cage [11]. The clinical and radiological outcomes of the hollow cylindrical n-HA/PA66 cages used in ACDF for the treatment of CDDD patients were suggested to be satisfactory in both short- and long-term follow-up [9, 12]. However, the n-HA/PA66 cage has relatively high subsidence rates (10.6%) during long-term follow-up, which could lead to cervical kyphosis, neural foramen stenosis, and yellow ligament folds after surgery [9, 10].

**Fig. 1:**
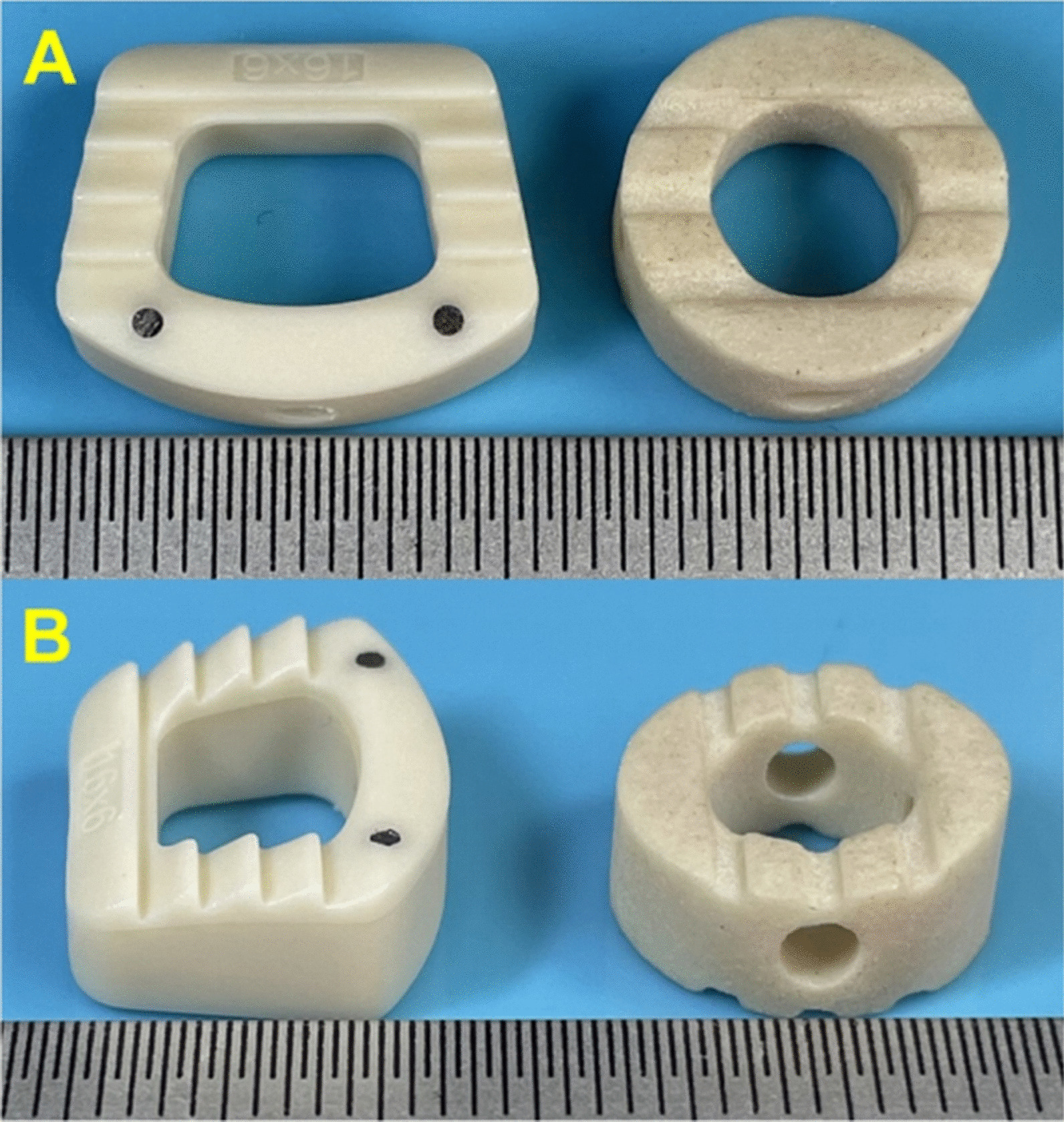
Horizontal view (**a**) and lateral view (**b**) of the novel n-HA/PA66 cage and the hollow cylindrical n-HA/PA66 cage

Previous studies have demonstrated that the morphology of the cage might be one of the most important factors related to the incidence of subsidence [13–15]. Hence, to decrease the incidence of subsidence, the morphology of the n-HA/PA66 cage was redesigned (the novel n-HA/PA66 cage). The novel n-HA/PA66 cage is a horseshoe-shaped cage with a more suitable shape and larger bone graft volume than the hollow cylindrical n-HA/PA66 cage (Fig. 1). Nevertheless, to our knowledge, no studies have compared the subsidence rates of the novel n-HA/PA66 cage and the hollow cylindrical n-HA/PA66 cage. Therefore, the purpose of this study was to compare the long-term clinical and radiological outcomes of the novel n-HA/PA66 cage with the hollow cylindrical n-HA/PA66 cage after ACDF for the treatment of single-level CDDD.

## Materials and methods

This study was a retrospective study that was approved by the ethics committee of West China Hospital of Sichuan University, and informed consent was obtained from the patients. All methods were carried out in accordance with relevant guidelines and regulations.

Fifty-two patients with CDDD who underwent single-level ACDF with the novel n-HA/PA66 cage (Group A) in our department between January 2013 and July 2015 were included. Fifty-five patients with hollow cylindrical n-HA/PA66 cages (Group B) placed after ACDF were also included for comparison. The inclusion criteria were as follows: (1) radiculopathy and/or myelopathy from single-level cervical disk herniation, (2) no response to 3 months of nonsurgical management, and (3) no previous spine surgery. The exclusion criteria were (1) previous spine surgery, (2) active infection, and (3) inflammatory spondyloarthropathies. All patients were followed up for at least 7 years.

All surgeries for all patients in this study were accomplished by the same surgeon and by following standard surgical procedures [4, 16]. All procedures were performed in supine position and through a transverse skin incision on the right side of the neck. After resecting the cartilage of the endplates at the operation level and removing the osteophytes as well as the ruptured posterior longitudinal ligament, a suitable cage filled with morselized bone from local decompression was inserted into the intervertebral space. The operated level was then stabilized by an ATLANTIS Anterior Cervical Plate System (Medtronic Sofamor Danek USA, Inc. Memphis, TN). All patients were told to wear a cervical collar for 6 weeks postoperatively.

**Fig. 2:**
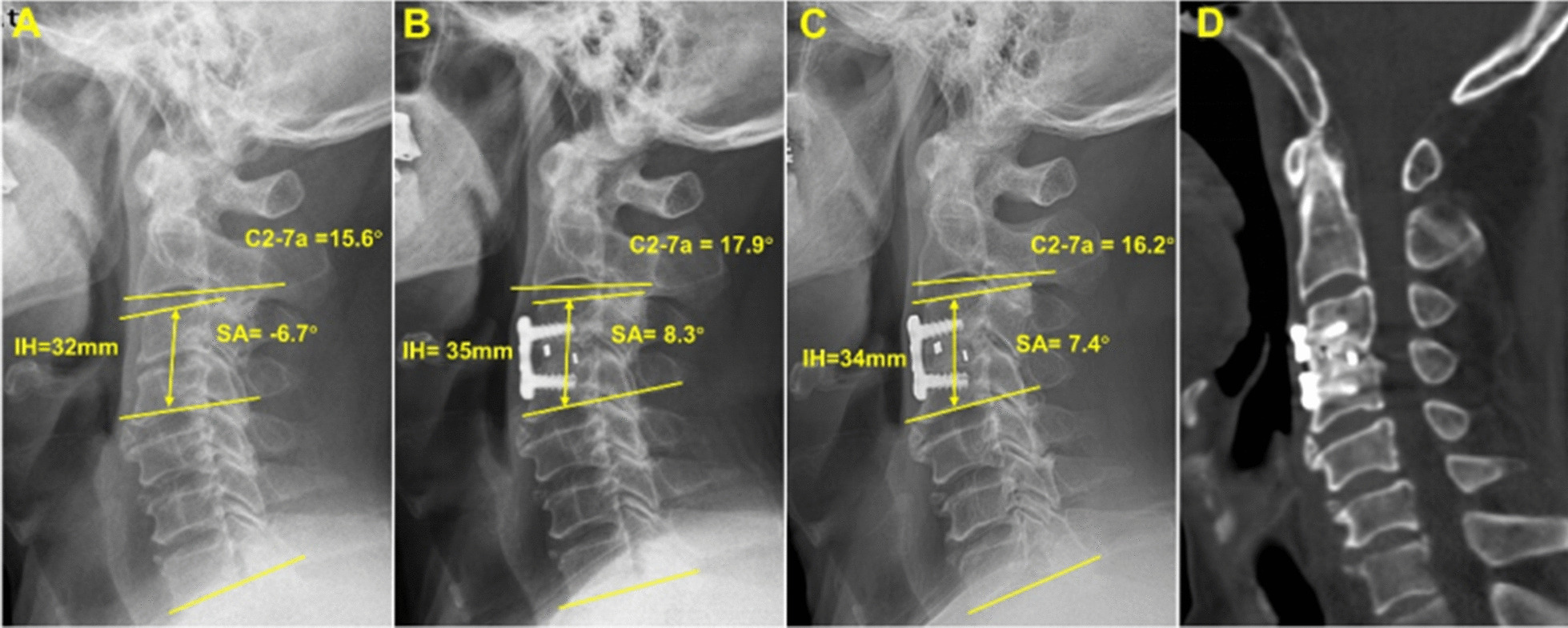
A 67-year-old man underwent ACDF with a novel n-HA/PA66 cage. **a** Preoperative lateral radiograph. **b** Lateral radiograph at 6-month post-surgery. **c** Lateral radiograph at the final follow-up. **d** 3d-CT at the final follow-up

Frontal and lateral radiographs and three-dimensional CT (3d-CT) of the cervical spine were obtained preoperatively, 6 months postoperatively, and at final follow-up (Fig. 2). The following parameters obtained from lateral radiography were used to evaluate the radiological outcomes: intervertebral height (IH); the distance from the midpoint of the superior endplate of the upper vertebrae to the midpoint of the inferior endplate of the lower vertebrae; C2-7 angle (C2-7a); the Cobb angle between the C2 and C7 vertebrae; segmental alignment (SA); and the Cobb angle between the upper and lower vertebra of the implanted level. Negative values were defined as kyphosis, while positive values were defined as lordosis. The loss of IH > 2 mm was regarded as subsidence [9]. The fusion status was evaluated on 3D-CT by the 5-grade criteria proposed by Brantigan and Steffee [17]: grade 4 or 5 were defined as fused; Grade 3 was defined as uncertain; Grade 1 or 2 were defined as unfused.

The clinical outcomes were evaluated by the visual analog scale (VAS) and Japanese Orthopedic Association (JOA) scores preoperatively, 6 months postoperatively, and at the final follow-up. We also collected the patient satisfaction levels at the final follow-up: very satisfied, somewhat satisfied, somewhat dissatisfied, and very dissatisfied [5].

Statistical analysis was performed by using SPSS 22.0 (IBM Corp., Armonk, NY, USA). Quantitative variables were expressed as the mean ± standard deviation and analyzed by Student’s t test or the Mann‒Whitney U test as appropriate. The χ^2^ test or Fisher’s exact test was used to analyze categorical variables. Statistical significance was set at *P* < 0.05.

## Results

The average follow-up time of the 107 included patients was 96.1 ± 7.5 months. The demographic data (age, sex, smoking status, operative time, and blood loss) of the patients in Group A and Group B were comparable (Table 1). In this study, the operative segments included C3/4, C4/5, C5/6, and C6/7. The difference in the proportions of the different operative segments between Group A and Group B was not significant (Table 1).

**Table 1:** Patient demographic data

	Group A (*n* = 52)	Group B (*n* = 55)	*P*
Age (years)	53.0 ± 8.7	52.8 ± 6.5	0.882
Gender (male/female)	30/22	29/26	0.606
Smoker	11/52	14/55	0.599
Operative time (min)	95.5 ± 18.5	92.8 ± 15.3	0.413
Blood loss (mL)	84.1 ± 54.4	81.5 ± 28.1	0.612
*Segments*			0.524
C3/4	8	6	
C4/5	10	12	
C5/6	17	18	
C6/7	17	19	
Follow-up time	95.2 ± 6.5	96.8 ± 8.2	0.268

The radiological outcomes of the patients in Group A and Group B are listed in Table 2. The SA and C2-7a of the patients in both groups were significantly improved at 6 months postoperatively and at the final follow-up. No significance was found regarding pre- and postoperative SA and C2-7a between Group A and Group B. The fusion rates at 6 months postoperatively (57.6%) and final follow-up (98.0%) in Group A were similar to those in Group B (56.3% at 6 months postoperatively and 96.3% at final follow-up, respectively). The preoperative and 6-month postoperative IHs in Group A and Group B were comparable. However, the IH at the final follow-up in Group B was significantly smaller than that in Group A (35.9 mm vs. 36.7 mm). No subsidence was observed at 6 months follow-up in either group, but the difference in the subsidence rates between Group A (5.8%, 3/52) and Group B (18.2%, 10/55) at the final follow-up was significant (*P* < 0.05).

**Table 2:** Radiological outcomes

	Group A (*n* = 52)	Group B (*n* = 55)	*P*
*SA (°)*			
Pre-op	2.0 ± 3.6	1.6 ± 3.3	0.556
6 m post-op	8.6 ± 3.3*	8.2 ± 3.7*	0.563
Final follow-up	7.0 ± 3.3*^#^	6.6 ± 2.9*^#^	0.513
*IH (mm)*			
Pre-op	35.4 ± 2.3	36.2 ± 2.8	0.119
6 m post-op	37.5 ± 2.1*	37.8 ± 2.5*	0.519
Final follow-up	36.7 ± 1.8*^#^	35.9 ± 1.9^#^	0.037
*C2-7a (°)*			
Pre-op	12.8 ± 9.6	13.1 ± 10.0	0.863
6 m post-op	20.5 ± 11.5*	19.3 ± 11.8*	0.609
Final follow-up	19.8 ± 9.4*	18.9 ± 10.0*	0.623
*Fusion rate*			
6 m post-op	57.6% (30/52)	56.3% (31/55)	0.890
Final follow-up	98.0% (51/52)	96.3 (53/55)	0.592
*Subsidence rate*			
6 m post-op	0% (0/52)	0% (0/55)	–
Final follow-up	5.8% (3/52)	18.2 (10/55)	0.046

SA, sagittal alignment; IH, intervertebral height; C2-7a, C2-7 angle; Pre-op, preoperative; 6 m post-op, 6-month postoperative

*p < 0.05 compared with pre-op

^#^*p* < 0.05 compared with 6 m post-op

Table 3 shows the clinical outcomes of the patients in Group A and Group B. The JOA and VAS scores of the patients in both groups were relieved postoperatively and were further improved at the final follow-up. No significant differences were found in the VAS score and JOA score of the patients in Group A and Group B during the different periods (preoperatively, 6 months postoperatively, and at the final follow-up). The proportion of patients who were very satisfied or somewhat satisfied in Group A was 90.4% (47/52), which was similar to that in Group B (89.1%, 49/55).

**Table 3:** Clinical outcomes of the patients

	Group A (*n* = 52)	Group B (*n* = 55)	*P*
*JOA score*			
Pre-op	8.1 ± 1.3	8.6 ± 7.7	0.094
6 m post-op	13.6 ± 2.2*	13.9 ± 1.8*	0.350
Final follow-up	14.2 ± 1.6*#	14.5 ± 1.6*#	0.351
*VAS score*			
Pre-op	8.1 ± 1.1	7.7 ± 1.4	0.188
6 m post-op	5.3 ± 0.9*	5.1 ± 1.3*	0.332
Final follow-up	2.0 ± 0.7*#	1.7 ± 0.8*#	0.084
*Patients’ satisfaction*			0.995
very satisfied	14	15	
somewhat satisfied	33	34	
somewhat dissatisfied	4	5	
very dissatisfied	1	1	

JOA, Japanese Orthopedic Association; VAS, visual analog scale; Pre-op, preoperative; 6 m post-op, 6-month postoperative

*p < 0.05 compared with pre-op

^#^*p* < 0.05 compared with 6 m post-op

## Discussion

HA is the main inorganic phase of the bone matrix, so it is widely applied to make scaffolds for bone tissue repair [18]. PA66 is a form of opalescent crystalline polymer with excellent mechanical properties (good elasticity, strength, and high elongation), and its unique molecular structure is beneficial for its modification [19]. The nHA crystals and the carboxy and amide groups of PA66 are similar to bone apatite and collagen, respectively, so the n-HA/PA66 composites were used to mimic natural bone in previous studies. They found that the physical, chemical, and mechanical characteristics of n-HA/PA66 match well with those of natural bone [11]. The biocompatibility and oseogenesis of the n-HA/PA66 composite scaffolds were also indicated to be good both in vitro and in vivo [20].

The n-HA/PA66 cage used in ACDF was firstly designed as a hollow cylindrical shape and some scholars has reported its clinical and radiological outcomes. Yang et al. [12] investigated the outcomes for the use of hollow cylindrical n-HA/PA66 cages for single-level ACDF and concluded that the hollow cylindrical n-HA/PA66 cage is a satisfactory reconstructing implant after ACDF. Hu et al. [9] further analyzed the long-term follow-up (> 7 years) results of hollow cylindrical n-HA/PA66 cages used in single-level ACDF in comparison to PEEK cages. Although there were similar radiological and clinical outcomes with the hollow cylindrical n-HA/PA66 cage as the PEEK cage, the subsidence rates of the hollow cylindrical n-HA/PA66 cage were relatively high (10.6%). The authors speculated that the main reason may be that the shape of the hollow cylindrical n-HA/PA66 cage was less suitable than that of the PEEK cage, which had an impact on the biomechanical properties of the cage.

Sequentially, to reduce the subsidence rate, the hollow cylindrical n-HA/PA66 cage was redesigned into a horseshoe-shaped cage with a more suitable shape and larger bone graft volume (novel n-HA/PA66 cage). In this study, 52 patients with novel n-HA/PA66 cages (Group A) and 55 patients with hollow cylindrical n-HA/PA66 cages (Group B) were included. The 6-month postoperative IHs in both groups were comparable and significantly improved after the surgery. However, the IH at the final follow-up of Group A was significantly higher than that of Group B (36.7 mm vs. 35.9 mm, *p* < 0.05). Meanwhile, the subsidence rate at the final follow-up of Group A was 5.8% (3/52), which was significantly lower than that of Group B (18.2%, 10/55). These results indicated that the modifications to the morphology of the hollow cylindrical n-HA/PA66 cage were effective in lowering the subsidence rate.

Subsidence is associated with multiple factors, such as bone mineral density, smoking, operative segments, and cage characteristics (material, shape, height, etc.) [9, 21]. In the present study, the patient demographic data (age, sex, operative time, blood loss, and segments) of Groups A and B were similar, while the primary difference between the two groups was cage shape. Different cage shapes correlated with different cage-endplate interfaces and bone graft shapes and volumes and finally led to variational postoperative fusion and subsidence resistance [14, 22]. Compared to the hollow cylindrical n-HA/PA66 cage, the novel n-HA/PA66 cage’s horseshoe shape has more inverse distributed jags, which can better mimic the concave contour of the vertebral endplate and improve the contact area and anchorage. Interestingly, the bone graft volume of the novel n-HA/PA66 cage was higher than that of the hollow cylindrical n-HA/PA66 cage, but the fusion rates at 6 months postoperatively and at the final follow-up in both groups were comparable.

Postoperative complications such as cervical kyphosis, recurrence of neurological symptoms, and internal fixation failure may be related to cage subsidence because it can compromise IH and neural foramen stenosis [9, 10]. Fortunately, no patients in either group complained about subsidence-related symptoms during the follow-up. Furthermore, the JOA and VAS scores in Groups A and B were similar; they improved postoperatively and were maintained at the final follow-up. These findings could explain the comparably ideal patient satisfaction at the final follow-up in both groups.

There were several limitations in the present study. First, this was a retrospective study with a small sample size. Second, this study did not analyze patients with multiple CDDD. Hence, future prospective studies with more patients with multiple CDDD are required to investigate the outcomes when using novel n-HA/PA66 cages.

## Conclusions

The novel n-HA/PA66 cage achieved favorable SA, C2-7a, fusion rate, and clinical outcomes, which were similar to those of the hollow cylindrical n-HA/PA66 cage in patients with CDDD who underwent single-level ACDF during a 7-year follow-up. At the final follow-up, the patients with novel n-HA/PA66 cages had a lower subsidence rate and higher IH than the patients with hollow cylindrical n-HA/PA66 cages.

## Data Availability

Data will be available upon request to the corresponding author.

## Abbreviations

n-HA: Nanohydroxyapatite
PA: Polyamide
ACDF: Anterior cervical discectomy and fusion
CDDD: Cervical degenerative disk disease
IH: Intervertebral height
C2-7a: C2-7 angle
SA: Segmental alignment
VAS: Visual analog scale
JOA: Japanese Orthopedic Association
PEEK: Polyetheretherketone
3d-CT: Three-dimensional CT
TLIF: Transforaminal lumbar interbody fusion
ACCF: Anterior cervical corpectomy and fusion
